# An Emerging Problem of Shisha Smoking among High School Students in Ethiopia

**DOI:** 10.3390/ijerph18137023

**Published:** 2021-06-30

**Authors:** Selamawit Hirpa, Andrew Fogarty, Adamu Addissie, Linda Bauld, Thomas Frese, Susanne Unverzagt, Eva Johanna Kantelhardt, Sefonias Getachew, Wakgari Deressa

**Affiliations:** 1 Department of Preventive Medicine, School of Public Health, Addis Ababa University, Addis Ababa 9086, Ethiopia; adamuaddissie@gmail.com (A.A.); safoget@yahoo.com (S.G.); deressaw@gmail.com (W.D.); 2 Institute of General Practice and Family Medicine, Center of Health Sciences, Martin-Luther-University Halle-Wittenberg, 06097 Halle (Saale), Germany; thomas.frese@uk-halle.de (T.F.); susanne.unverzagt@uk-halle.de (S.U.); 3 NIHR Biomedical Research Centre, Division of Epidemiology and Public Health, University of Nottingham, Clinical Science Building, Nottingham City Hospital, Nottingham NG5 1PB, UK; Andrew.Fogarty@nottingham.ac.uk; 4 Institute for Medical Epidemiology, Biostatics and Informatics, Center of Health Sciences, Martin-Luther-University Halle-Wittenberg, 06097 Halle (Saale), Germany; eva.kantelhardt@uk-halle.de; 5 Usher Institute and SPECTRUM Consortium, University of Edinburgh, Edinburgh EH8 9AG, UK; Linda.Bauld@ed.ac.uk

**Keywords:** shisha smoking, high school students, Ethiopia

## Abstract

Shisha smoking is also known as hookah, water pipe, goza, and nargile. Shisha use among the young is increasing globally. Shisha smoke results in a high concentration of carbon monoxide, tar, nicotine, and heavy metals which can be toxic to humans, especially with chronic exposure. This study aims to determine the prevalence and risk factors of shisha smoking among in-school adolescents in Ethiopia. Four regional states in Ethiopia (Oromia, Amhara, Southern Nations, Nationalities, and Peoples’ Region, Tigray) and the capital city (Addis Ababa) were the study areas. A two-stage cluster sampling approach was employed to produce a representative sample. From the sampling frames in the study areas, 36 high schools were selected randomly. A multi-level logistic regression analysis was used to account for cluster-specific random effects, the effect of individuals’, and school-level variables for ever-use of shisha. A total of 3355 secondary school grade 9 and 10 students aged between 13 and 22 years took part in this study. A total of 86 (2.6%) and 20 (0.6%) of the study participants, reported that they had ever smoked or were current smokers of shisha, respectively. Of all study participants, 38.6% perceived shisha as less harmful than cigarettes and 48.5% reported that they do not know which was more harmful to health. Students were more likely to ever use shisha if they had friend/s who smoke shisha (AOR = 16.8, 95% CI: 6.4–44.3), ever smoked cigarettes (AOR = 8.2, 95% CI: 3.4–19.8), ever used khat (AOR = 4.2, 95% CI: 1.9–10.4), ever used marijuana (AOR = 3.9, 95% CI: 1.4–11.1), ever used smokeless tobacco (AOR = 3.1 95% CI: 1.1–8.4), and students had received income from their parents (AOR = 3.1 CI: 1.1–8.8). Prevalence of ever and current use of shisha among high school students is low in Ethiopia compared to many countries in Africa. The majority of adolescents perceived shisha as less harmful to health than cigarette smoking. Health education about the harmful effects of shisha should be delivered to adolescents, along with information on other substances like khat, cigarettes, marijuana, and smokeless tobacco to prevent initiation of substance use.

## 1. Background

Tobacco is manufactured and used in different forms that include cigarettes, cigars, chewable tobacco, snuff, bidis, and shisha smoking, which is also known as hookah, water pipe, goza, and nargile [1]. Shisha is a flavored or non-flavored product that is smoked through a long hose where the smoke passes through water before reaching the smoker and the syrup tobacco content includes molasses, honey, vegetable glycerol, and fruit flavors [2].

In most parts of the world, cigarette smoking is considered the most common form of tobacco use, though shisha is becoming extensively used worldwide with high prevalence in the Middle East, Africa, and Asia [3].

Shisha smoke delivers high concentrations of carbon monoxide, nicotine, tar, and heavy metals at levels that are as high as or higher than a cigarette smoke and which can be toxic to humans, especially with chronic exposure [4,5]. Shisha smoking has been associated with different health problems like oral and lung cancer, cardiovascular, and respiratory diseases [6,7].

A study conducted in the USA on adolescents aged 12–17 years showed that the health effects of shisha smoking were not well understood by young people, and the perceived risk to health was less than for cigarette smoking [8]. Another study conducted in Saudi Arabia among secondary school students found that shisha was considered less harmful than cigarettes by 47.2% of study participants and more socially accepted than cigarettes [9]. In the same study, almost 60% of adolescents presumed that harmful substances would be filtered out through water smoking shisha [9].

Shisha use among young people is increasing globally [10]. Based on studies conducted in Africa, the prevalence of ever use of shisha among youth ranges from 3% in Ghana [3] to 26% in Rwanda [11]. A study conducted at Debre Berhan University in Ethiopia found that 4.2% of students had ever used shisha [12]. Studies reported that peer influence, regular alcohol drinking, having a friend/s who smokes shisha, being aware of shisha cafes availability, and a positive attitude towards shisha use were associated with shisha smoking [11,13].

Ethiopia is one of several countries in sub-Saharan Africa with strong tobacco control laws. Based on the Ethiopian Food and Drug Administration Control Proclamation Number 1112/2019, selling and smoking shisha is forbidden [2]. However, very limited evidence exists regarding tobacco product use among adolescents in the country. Almost all existing studies focus on cigarette smoking. The aim of this study was to determine the prevalence of shisha smoking among high school students in Ethiopia. We also examined potential factors associated with shisha smoking. The results will be used to engage with policymakers about the extent of the problem and risk factors.

## 2. Methods

### 2.1. Study Design and Setting 

This cross-sectional study was conducted in Ethiopia, a country comprised of 10 regional states and two city administration councils. The business capital, Addis Ababa city, and the business capitals of the four big regions (i.e., Adama, Bahir Dar, Hawassa, and Mekelle) with their respective peri-urban districts were involved in the study. In Ethiopia, secondary education has two cycles; the first cycle covering grades 9 and 10 and the second cycle covering grades 11 and 12. The majority of secondary school students are aged between 15 and 18 years.

There were 301 urban and 96 peri-urban high schools in the study areas. The total number of high school students in the study regions and their respective peri-urban districts were; 130,619 in Addis Ababa, 43,575 in Adama, 39,670 in Bahir Dar, 36,816 in Hawassa, and 30,608 in Mekelle.

### 2.2. Study Population and Sample Size Calculation

Adolescents currently attending school aged 13–22 years who were in grades 9 and 10 participated in this study. The minimum sample size was calculated using OpenEpi Version 7.02 statistical software [14]. The sample size was powered to estimate the prevalence of cigarette, shisha, khat use, and alcohol consumption [15,16] with 95% precision, 80% power and considering an intra-cluster correlation coefficient (ICC) of 0.1, and a non-response rate of 30% among the students and a 10% rate from the schools. The total sample size was 4714 school going adolescents clustered into 50 schools.

### 2.3. Sampling Procedures

A two-stage cluster sampling approach was employed to produce a representative sample of students. A sampling frame was prepared separately for each urban and peri-urban study areas. A total of 50 schools were selected randomly from the list in all study areas. Because of the occurrence of COVID-19 pandemic at the end of data collection around mid-March 2020, it was not possible to collect data from all 50 selected schools. We were able to collect data in 36 schools. Subsequent to selecting the schools, one classroom from both grade 9 and 10 in the study school was selected. Then all the students in the selected classrooms were invited to participate in the study. Students who were absent from their class at the time of the survey, unable to fill the questionnaire with any health-related issues, and those who were not willing to participate in the survey were excluded.

### 2.4. Survey Instruments and Data Collection

The questionnaire was developed in English and later translated to three different local languages, Amharic, Afan Oromo, and Tigrigna and was translated back into English to check for consistency of the ideas across all the versions. The questionnaire was developed by considering shisha use related questions from the Global Youth Tobacco Survey (GYTS) instrument [17]. The tool was pre-tested among 250 students (100 students from Addis Ababa for Amharic, 100 students from Sebeta for Afan Oromo, and 50 students from Adigrat for the Tigrigna version of the questionnaire). The questions were modified by the first author based on this study objective and school environment questions were developed by the last author. All research team members reviewed the tool. Two-day data collection training was given to ten experienced data collectors. Research team members supervised the data collection. Data were collected from 9 to 17 March 2020. The questionnaires were distributed to grade 9 and 10 students in the selected schools and classes. Students were asked to complete the questionnaire individually and to consult the data collectors for any ambiguity on the questions. Also, data were collected from all school directors in regard to the availability and feasibility of shisha smoking in school environment areas.

### 2.5. Data Processing and Analysis 

Data were checked for completeness and consistency before and during data entry that was conducted using Epidata Version 3.1 software [18]. Data were analyzed using Stata/SE 14.0 software [19]. Frequency and percentages of study participants and school-level characteristics were calculated. The prevalence of current and ever use of shisha was estimated. In the bivariable analysis, the chi-square test was used to test the significance of the association between shisha use and the individual level characteristics. Mean and standard deviation were used to summarize continuous variables. The primary outcome variable of this study is ever use of shisha. Independent variables in the level-1 model are age, sex, parent’s residence, source of income, ever smoked cigarette, ever used smokeless tobacco, ever drank alcohol, ever used khat, ever used marijuana, friends use shisha, and shisha smoking more harmful to health compared to cigarette. Independent variables in model-2 included all the 11 variables in model-1 and an additional 4 school level variables (school type, shisha houses within 100 m radius from the school compound, teacher in the school who smokes shisha, and the number of students in the school).

In this study, ever use of shisha was defined as shisha smoking at least once in a lifetime, current shisha smoker as shisha smoking at least once in the past 30 days from the study period and a peri-urban area as a town within 50 km radius from the main city in the specific region.

A two-level mixed-effects logistic regression model was fitted to adjust for confounding variables. The model accounted for the effects of student-level characteristics (level-1) and school (level-2) factors on ever use of shisha. The model fitting was a three-step process where the null-model (model-0) measured the random effect and intra cluster correlation (ICC) in the odds of ever use of shisha. The ICC represented the proportion of the between-schools variation on ever use of shisha in the total variation [20]. The total variation is, between schools plus the within school variation of the chances of ever use of shisha. The first model (model-1) was built to assess the effect of student level predictors on the odds of ever use of shisha. The final model (model-2) was built to assess the effect of student-level characteristics and cluster level (school level) factors on ever use of shisha. We used the xtmelogit command in Stata to perform the multi-level mixed effect logistic regression analyses. Multicollinearity was checked for independent variables before the model was fitted and none were dropped from the model as all the variables had a variance inflation factor (VIF) value of <10 [21]. Crude odds ratios (ORs) and adjusted ORs with the corresponding 95% confidence intervals (CIs) were estimated using univariate logistic and multivariate mixed-effects logistic regression models, respectively, to quantify the associations between potential predictors and outcome variable. The level of statistical significance was set at a *p*-value < 0.05.

## 3. Results

### 3.1. Socio Demographic Characteristics 

Data were collected from 36 schools (11 private and 25 governmental schools), 82% of the targeted 50 schools. The total number of grade 9 and 10 students per school ranged from 108 to 4191. Due to the COVID-19 pandemic, schools were closed suddenly in March, 2020. For this reason, it was not possible to finalize the data collection and collect an equal amount of data from all regions. From a total of 3457 students who were invited to this study, 3355 (97%) participated. After discarding eight questionnaires that were incomplete for the majority of important questions, data from 3347 participants remained in the analysis. The age of 2499 (74.7%) of the secondary school students ranged from 15–17 years (Table 1). The mean age (±SD) was 16.5 ± 1.4 years and almost 54% of them were females. In this study, 2618 (78%) of study participants were from government schools. More than 3000 (90%) students got their income from their parents.

### 3.2. Shisha Use Practice and Perception about Shisha Smoking 

Of a total of 3347 students, 86 (2.6%) and 20 (0.6%) of the study participants reported that they had ever smoked or were current smokers of shisha, respectively (Table 2). The peri-urban areas of Hawassa had the highest proportion of ever use of shisha (7.1%), followed by Addis Ababa (4.8%), and Adama (2.6%) cities. The region has a significant association with ever use of shisha (*p*-value < 0.001). Sixty-seven (4.4%) male and 19 (1.1%) female participants ever smoked shisha. Of all study participants, 1285 (38.4%) perceived shisha as less harmful for health than tobacco while 1616 (48.3%) reported that they did not know about this. The perception about harmful effects of shisha has significant association with ever use of shisha (*p*-value < 0.001).

### 3.3. High School Students’ Parents and Friends’ Shisha Smoking Practice 

Two hundred and five (6.1%) students reported that their friends smoke shisha while 526 (15.7%) were not sure or did not know their status (Table 3). In more than 90% of the study participants, neither their mothers nor fathers smoke shisha, but 205 (6.1%) participants reported that at least one of their friends smokes shisha.

### 3.4. School Environments and Shisha Smoking 

A total of 251 (7.5%) school-going adolescents mentioned that there were houses for shisha smoking within 100 m from the school (Table 4). Of them, 113 (45%) reported that students smoke shisha in these houses. Sixteen (44.4%) of 36 school directors (who were school directors for 3347 students), responded that there were shisha houses within 100 m from the school compound. Moreover, it was reported that from 2 (5.6%) of these schools there were 2–3 teachers who smoke shisha.

### 3.5. School and Individual Level Factors for Ever Use of Shisha 

There was 36% variability between clusters (schools) with regards to the outcome variable ever use of shisha (ICC = 0.36, 95% CI: 0.2–0.6). Significantly associated variables with ever use shisha in model-1 and model-2 were similar (Table 5). Students were more likely to ever use shisha if they had friend/s who smoke shisha (AOR = 16.8, 95% CI: 6.4–44.3), ever smoked cigarettes (AOR = 8.2, 95% CI: 3.4–19.8), ever used khat (AOR = 4.2, 95% CI: 1.9–10.4), ever used marijuana (AOR = 3.9, 95% CI: 1.4–11.1), had ever used smokeless tobacco (AOR = 3.1 95% CI: 1.1–8.4), and students who had received income from their parents (AOR = 3.1 CI: 1.1–8.8). No significant association was shown for other characteristics of the students (age, sex, parent’s residence, perception about the health effects of shisha, and ever drink of alcohol) and school-level variables (school type, shisha houses near school, having shisha smoker teacher, and the number of students).

## 4. Discussion

We found a prevalence 2.6% of ever use of shisha and 0.6% current shisha smoking among adolescents attending high schools in Ethiopia. While these rates are low, they remain a concern, given the health harms associated with shisha smoking in a country that has a very low prevalence of tobacco use. Independent risk factors for ever-shisha smoking were having friends who smoke shisha, ever use of cigarettes, khat, marijuana, smokeless tobacco, and students who receive pocket money from their parents. Students’ age, sex, and perception about the health effects of shisha were not significantly associated with the outcome variable. School type (government or private), and shisha shops within 100 m from the school compound were not significantly associated with ever use of shisha.

This study is the first-ever conducted to describe shisha consumption among school-going adolescents covering a wide geographical area and large sample size in Ethiopia. It included a wide range of school-going adolescents from the main capital city, urban, and peri-urban areas of the four different regions. The response rate was very high, at 97%, which decreases the risk of bias due to differential responses.

Our study has limitations. First, the cross-sectional study design cannot identify a cause-effect relationship. Secondly, our study finding is limited in the generalizability to the rural population in Ethiopia and for adolescents in the same age group who do not attend school. Third, the period of data collection coincides with the COVID-19 pandemic when schools’ closures prohibited the finalization of data collection and limited the final sample size. Finally, social desirability bias might be a potential limitation of this study. However, school directors and teachers were not present in the classroom at the time of the data collection. The selected class section was divided into two for increasing the privacy of the students when completing the self-administered questionnaire. 

The prevalence of ever and current use of shisha in our study was low compared to studies conducted in African countries including Ethiopia. Our study findings are comparable to the Global Youth Tobacco Survey (GYTS) conducted in Ghana that reported 3.1% of ever use of shisha in the age group of 11–17 years [3]. A higher prevalence of shisha use was reported in African countries like Rwanda (26.1%) where private university students participated and a study in Sudan among high school students reported 13.4% [11,22]. This could be explained by the fact that these studies were conducted in capital cities where shisha smoking practice might be higher.

A study in Bale (Southeast, Ethiopia) conducted among high school students showed that 5.6% were current users of shisha [23]; the study included only one specific area and khat chewing in this area was a common practice which might explain the higher prevalence of shisha use. A study among high school students in Addis Ababa and surrounding rural areas reported that 7.1% were ever use and 0.8% were current users of shisha [15]. This could be explained by more schools included in this study being mainly from Addis Ababa and within a short distance (up to 165 km) from the capital city, Addis Ababa. Our study included only 6 schools from Addis Ababa and a representative number of schools were also included from the northern part of Ethiopia where shisha smoking practice was not common.

Based on this study, the Hawassa peri-urban area had the highest prevalence of ever smoking shisha which could be associated with the widespread practice of khat chewing in the area. A previous study in one of our study areas in the Hawassa peri-urban area showed that the high expansion of khat due to its economic advantage is superior to all other crops grown in the area [24]. Addis Ababa and Adama followed in a higher proportion of ever use of shisha where almost all schools were taken from the main cities. Based on this study, students who received pocket money from parents were more likely to ever use shisha. Obtaining a regular allowance from parents may provide an opportunity to students to have extra money, with implications for students to be exposed to using different substances.

This study found that 2732 (81.6%) students perceived shisha as harmful to health. Over 39% of the study participants mentioned that it was less harmful than a cigarette, and almost 50% stated that they do not know whether it is as harmful as cigarettes or not. This indicates that there was low awareness about the health effects of shisha smoking among school going adolescents. In Ethiopia, it is not common to hear discussion of the health effects of shisha smoking. There are myths about shisha smoking that it is good for skin color and harmful substances would be filtered out through the water. Similar to our study finding, a systematic review of global-north and south studies reported that the majority of university students could identify the health hazards of shisha smoking and most students identified shisha as less harmful than cigarettes [25].

Our study indicated that there were shisha shops within a 100 m radius for 7.5% of the schools and some students reported smoking shisha in these houses. Based on Ethiopian Food and Drug Administration Control Proclamation 1112/2019, it is forbidden to smoke and sell flavored tobacco products [2]. Moreover, it is forbidden to sell any kind of tobacco products within 100 m of a school. Our study indicated that there are gaps in the enforcement of this tobacco control law in Ethiopia. Similarly, a recent report identified an expansion of shisha cafes in Addis Ababa [2].

Having a friend who smokes shisha was significantly associated with ever use of shisha in our study. This finding is supported by the fact that adolescents are influenced by their peer groups. In agreement with our finding, studies conducted among young adolescents in Saudi Arabia, Gambia, and Sudan showed that having friends who smoke shisha is a risk factor for the ever/current use of shisha smoking [9,22,26]. Another study conducted in different areas of Ethiopia (Debre Berhan, Shashemene, and Bale) among youth, reported that those with friends who use any kind of substance were more likely to be users of the same substances [12,23,27].

Student level factors such as ever use khat, marijuana, cigarettes, and smokeless tobacco were significantly associated with ever use of shisha. A systematic review discussed that khat chewing is associated with using tobacco products among high school students [28]. Moreover, a study conducted in the eastern part of Ethiopia showed that khat chewing is associated with any kind of tobacco products including shisha [29]. This association might be explained by the enhanced effect of tobacco products and khat chewing [30]. Khat users might interchangeably use cigarettes or shisha to complement the effect of khat. Furthermore, once students are exposed to using one substance there is a probability that they may be exposed to multiple substances.

## 5. Conclusions 

Shisha smoking practice among high school students in Ethiopia is low compared to many countries in Africa. However, shisha use is becoming a global challenge and is linked with the use of other addictive substances including cigarette smoking. Preventive programs should be in place to increase awareness among students of the negative health effects of shisha smoking. In addition, where tobacco control measures are already in place to limit the availability of the product, for example bans on shisha houses near schools, these should be properly enforced. Law that bans the selling of khat and marijuana to the young age group should be in place and enforced.

## Figures and Tables

**Table 1:** Study participants and school level characteristics in Ethiopia (March 2020).

Variable	Category	Frequency	Percentage (%)
Sex (*n* = 3319)	Female	1804	53.9
	Male	1515	45.3
Age (*n* = 3321)	13–14	99	3.0
	15	689	20.6
	16	1055	31.5
	17	755	22.6
	18	502	15.0
	19–20	190	5.7
	>20	31	0.9
Religion (*n* = 3313)	Orthodox Christian	2203	65.8
	Muslim	367	11.0
	Protestant	654	19.6
	Catholic	51	1.5
	Other	38	1.1
Living area (*n* = 3347)	Addis Ababa	458	13.7
	Adama	586	17.5
	Bahir Dar	875	26.2
	Hawassa	561	16.8
	Mekelle	867	25.9
School type (*n* = 3347)	Private	729	21.8
	Government	2618	78.2
Total number of grade 9 and 10 students in the school) (*n* = 36)	108–350	9	25.0
	408–980	7	19.4
	1038–1600	9	25.0
	1604–2273	6	16.7
	≥2677	5	13.9
Student grade level (*n* = 3347)	Grade 9	1685	50.3
	Grade 10	1662	49.7
Parents living area (*n* = 3310)	Rural	1185	35.4
	City	2125	63.5
Source of income (*n* = 3328)	Parents	3004	90.4
	Work for myself	257	7.7
	Other	67	2.0

**Table 2:** Prevalence and perception about shisha smoking among high school students in Ethiopia (March 2020).

Variable		Frequency (%)		*p*-Value
		Never Shisha Users	Ever/Current Shisha Users	
Ever used shisha by regions	Total	3245 (97.4)	86 (2.6)	<0.001
	Addis Ababa	432 (95.2)	22 (4.8)	
	Adama	568 (97.4)	15 (2.6)	
	Bahir Dar	870 (99.8)	2 (0.23)	
	Hawassa	520 (92.9)	40 (7.1)	
	Mekelle	855 (99.2)	7 (0.8)	
Ever used shisha	Male	1441 (95.6)	67 (4.4)	<0.001
	Female	1776 (98.9)	19 (1.1)	
Current use of shisha by regions	Total	3311 (99.4)	20 (0.6)	<0.001
	Addis Ababa	445 (98)	9 (2.0)	
	Adama	580 (99.5)	3 (0.5)	
	Hawassa	554 (98.9)	6 (1.1)	
	Bahir Dar	872 (100)	0 (0)	
	Mekelle	860 (99.8)	2 (0.2)	
Is shisha smoking harmful to health?	No	60 (84.5)	10 (14.1)	<0.001
	Yes	2677 (97.6)	55 (2)	
	I do not know	486 (96)	19 (3.7)	
Is shisha smoking less harmful than tobacco smoking?	No	357 (94)	23 (6)	<0.001
	Yes	1250 (97.3)	35 (2.7)	
	I do not know	1590 (98.4)	26 (1.6)	

**Table 3:** High school students’ parents and friends’ shisha-smoking experience in Ethiopia (March 2020).

Variable	Categories	Frequency (%)
Any of your friend’s smoke shisha (*n* = 3347)	No	2588 (77.3)
	Yes	205 (6.1)
	I am not sure	168 (5)
	I do not know	358 (10.7)
	Missing	28 (0.84)
How often do your friends smoke shisha (*n* = 205)	Every day	32 (15.6)
	Weekly	47 (22.9)
	Sometimes	71 (34.6)
	I am not sure	21 (10.2)
	I do not know	21 (10.2)
	Missing	13 (6.3)
Do any of your friends smoke shisha by region (*n* = 205)	Addis Ababa	90 (43.9)
	Adama	44 (21.5)
	Hawassa	29 (14.2)
	Bahir Dar	33 (16.1)
	Mekelle	9 (4.4)
Father smokes shisha (*n* = 3347)	Yes	11 (0.3)
	No	3041 (90.9)
	I do not know	126 (3.8)
	Not relevant for me	135 (4)
	Missing	34 (1)
Mother smokes shisha (*n* = 3347)	Yes	15 (0.45)
	No	3119 (93.2)
	I do not know	101 (3)
	Not relevant for me	67 (2)
	Missing	45 (1.3)

**Table 4:** Shisha smoking practice in the school environment in Ethiopia (March 2020).

Variable	Category	Frequency (%)
Any shop for shisha smoking within 100 m radius from the school compound (students’ responses) (*n* = 3347)	Yes	251 (7.5)
	No	1311 (39.2)
	I do not know	1754 (52.4)
	Missing	31 (0.93)
Students in the school smoke in these shisha shops (students’ responses) (*n* = 251)	Yes	113 (45.2)
	No	25 (10)
	I do not know	106 (42)
	Missing	7 (2.8)
Any shop house for shisha smoking within 100 m radius from the school (school director response) (*n* = 36)	Yes	16 (44.4)
	No	20 (55.6)
Grade 9 and 10 teachers from this school who smoke Shisha (school director response) (*n* = 36)	Yes	2 (5.6)
	No	34 (94.4)

**Table 5:** Multi-level logistic regression model for ever use of shisha among high school students in Ethiopia.

Variables		Ever Used Shisha (%) *n* = 86 (2.6%))	Model-1 OR (95% CI)	Model-2 AOR (95% CI)
Age	13–16	27 (31.4)	Reference	Reference
	17 and above	59 (68.6)	0.7 (0.3–1.6)	0.8 (0.32–1.8)
Sex	Male	67 (77.9)	Reference	Reference
	Female	19 (22.1)	0.5 (0.19–1.1)	0.5 (0.19–1.2)
Parent’s residence	Rural	25 (29.1)	Reference	Reference
	City	59 (68.6)	0.9 (0.3–2.4)	0.9 (0.31–2.5)
Source of income from parents	No	25 (29.1)	Reference	Reference
	Yes	61 (70.9)	3.3 (1.2–9.2) *	3.1 (1.1–8.8) *
Ever use of khat	No	29 (33.7)	Reference	Reference
	Yes	57 (66.3)	4.4 (1.9–10.4) *	4.2 (1.9–10.4) *
Ever use of smokeless tobacco	No	50 (58.1)	Reference	Reference
	Yes	35 (40.7)	3 (1.1–8.0)	3.1 (1.1–8.4)
Ever drink of alcohol	No	24 (27.9)	Reference	Reference 1
	Yes	62 (72.1)	1.4 (0.6–3.2) *	1.4 (0.61–3.4) *
Ever use of marijuana	No	54 (62.8)	Reference	Reference
	Yes	29 (33.7)	3.8 (1.4–11.0) *	3.9 (1.4–11.1) *
Ever smoked cigar rete	No	34 (39.5)	Reference	Reference
	Yes	52 (60.5)	9.2 (3.8–22.1) *	8.2 (3.4–19.8) *
Any of your friend smoke shisha	No	19 (22.1)	Reference	Reference
	Yes	49 (57)	16.7 (6.5–42.8) *	16.8 (6.4–44.3) *
Is shisha smoking less harmful than cigarette smoking	No	23 (26.7)	Reference	Reference
	Yes	35 (40.7)	1.3 (0.43–4)	1.5 (0.5–4.5)
	I do not know	26 (30.2)	1.1 (0.31–3.4)	1.1 (0.34–3.8)
School type	Government	64 (74.4)		1
	Private	22 (25.6)		3.9 (0.71–21.2)
Teachers in the school who smoke shisha				1
				4.7 (0.5–43.4)
Shisha houses within 100 m radius				1
				0.3 (0.07–1.3)
Number of students in the school				1 (0.99–1)

* Model-1—All relevant student level variables in relation to ever use of shisha use were included. * Model-2—The same student level variables and additional school level variables in relation to ever use of shisha were included.

## Data Availability

The datasets used and analyzed for the current study will be available from the corresponding author on reasonable request.

## Abbreviations

AOR	Adjusted odds ratio
APC	Article Processing Charge
CI	Confidence interval
GYTS	Global Youth Tobacco Survey
ICC	Intra-cluster correlation coefficient
IRB	Institutional review board
SNNPR	Southern Nations, Nationalities, and Peoples’ Region
SPH	School of Public Health
OR	Odds ratio
UKRI	UK Research and Innovations
VIF	Variance inflation factor
